# Application of multi-echo Dixon and MRS in quantifying hepatic fat content and staging liver fibrosis

**DOI:** 10.1038/s41598-023-39361-6

**Published:** 2023-08-02

**Authors:** Yanli Jiang, Jie Zou, Fengxian Fan, Pin Yang, Laiyang Ma, Tiejun Gan, Shaoyu Wang, Jing Zhang

**Affiliations:** 1 Second Clinical School, Lanzhou University, Lanzhou, People’s Republic of China; 2 Department of Magnetic Resonance, Lanzhou University Second Hospital, Cuiyingmen No.82, Chengguan District, Lanzhou, 730030 People’s Republic of China; 3 Gansu Province Clinical Research Center for Functional and Molecular Imaging, Lanzhou, People’s Republic of China; 4 MR Scientific Marketing, Siemens Healthineers, Shanghai, People’s Republic of China

**Keywords:** Liver fibrosis, Diagnostic markers

## Abstract

This study associated the liver proton density fat fraction (PDFF), measured by multi-echo Dixon (ME-Dixon) and breath-hold single-voxel high-speed T2-corrected multi-echo ^1^H magnetic resonance spectroscopy (HISTO) at 1.5 T, with serum biomarkers and liver fibrosis stages. This prospective study enrolled 75 patients suspected of liver fibrosis and scheduled for liver biopsy and 23 healthy participants with normal liver function. The participant underwent ME-Dixon and HISTO scanning. The agreement of PDFF measured by ME-Dixon (PDFF-D) and HISTO (PDFF-H) were compared. Correlations between PDFF and serum fat biomarkers (total cholesterol, triglyceride, and high- and low-density lipoproteins) and the liver fibrosis stages were assessed. PDFF were compared among the liver fibrosis stages (F0–F4) based on clinical liver biopsies. The Bland–Altman plot showed agreement between PDFF-D and PDFF-H(LoA, − 4.44 to 6.75), which have high consistency (ICC 0.752, *P* < 0.001). The correlations with the blood serum markers were mild to moderate (PDFF-H: r = 0.261–0.410, *P* < 0.01; PDFF-D: r = 0.265–0.367, *P* < 0.01). PDFF-D, PDFF-H, and steatosis were distributed similarly among the liver fibrosis stages. PDFF-H showed a slight negative correlation with the liver fibrosis stages (r = − 0.220, *P* = 0.04). Both ME-Dixon and HISTO sequences measured liver fat content noninvasively. Liver fat content was not directly associated with liver fibrosis stages.

## Introduction

Liver fibrosis is defined as excess deposition of extracellular matrix components such as collagens, glycoproteins, and proteoglycans in the liver. Liver fibrosis is a complex pathological process accompanied by steatosis, inflammation, and other changes. Liver fibrosis, a public health problem, is closely associated with various prevalent causes of chronic liver damage^1^. This response to liver damage is potentially reversible, so early diagnosis and accurate staging are highly important in clinical practice^2, 3^.

Liver steatosis is a pathological condition characterized by excessive fat deposition in the hepatocytes. It is the most common chronic liver disease, seen in viral hepatitis, genetic lipodystrophies, alcohol abuse, and more. If it has no competing causes, it is called nonalcoholic fatty liver disease (NAFLD)^4^, which can progress to more aggressive forms such as hepatitis, fibrosis, and cirrhosis^5^.

Recently, fast quantitative MRI techniques for acquiring proton density fat fraction maps within one breath-hold cycle have been introduced. Multi-echo Dixon (ME-Dixon) is a variant of 3D multi-gradient-echo sequences that allows fast imaging for simultaneous assessment of the fat fraction and transverse-relaxation time. It is a confounder-corrected chemical shift-encoded MRI technique that could potentially quantify total liver fat content. A breath-hold single-voxel high-speed T2-corrected multi-echo ^1^H MRS (HISTO) sequence can quantify lipid percentages by correcting for the R2 relaxations of water and fat. It is more accurate than traditional MRS in quantifying fat content. Recent studies have shown that these techniques could provide an accurate quantification of liver fat, verified by liver biopsies^6, 7^.

Liver fibrosis and steatosis are two independent but closely related pathological processes. Some research^3, 8, 9^ had pointed out the effect of steatosis on liver fibrosis. However, few studies have focused on the impact of liver fat content on the liver fibrosis stages. Therefore, this prospective study aimed to explore the association between liver fat content, assessed by the ME-Dixon and HISTO techniques, and the liver fibrosis stages.

## Results

**Figure 1:**
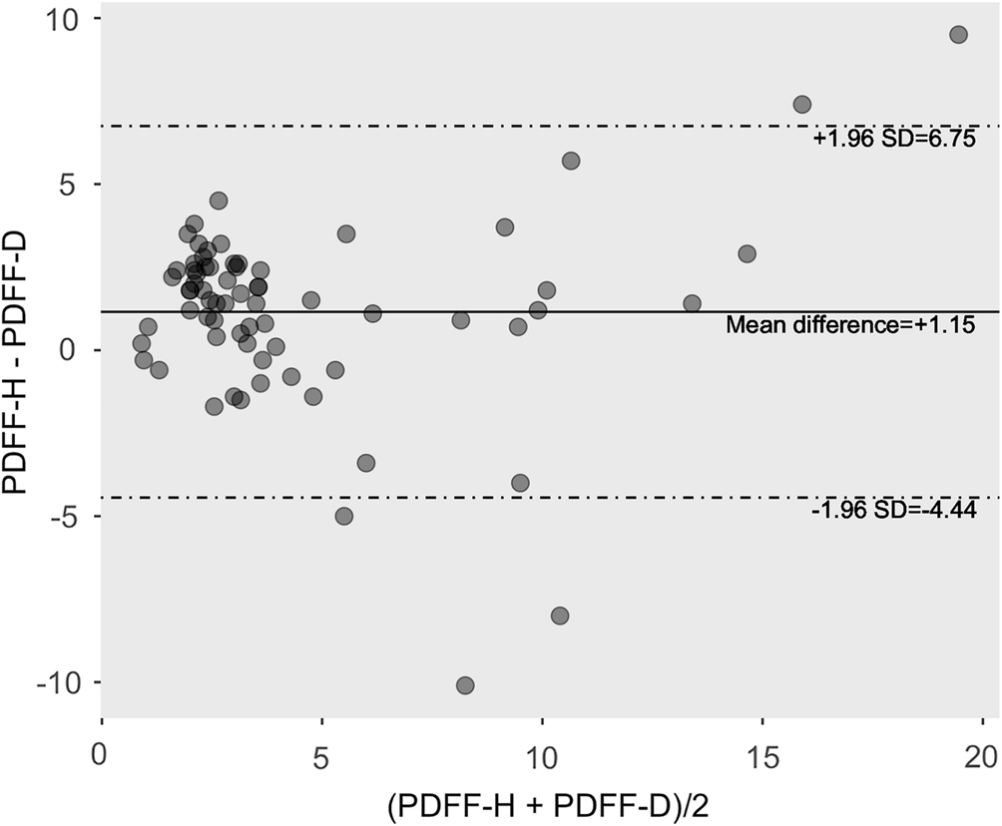
Bland–Altman plot for proton density fat fraction (PDFF), estimated using the breath-hold single-voxel high-speed T2-corrected multi-echo 1H MRS (HISTO) and multi-echo Dixon (ME-Dixon) sequences. The plot shows the − 1.96 and + 1.96 SD limits of the agreement (dotted lines) around the mean difference. *PDFF-D* PDFF estimated by ME-Dixon, *PDFF-H* PDFF estimated by HISTO.

The Bland–Altman plot for the PDFF agreement is shown in Fig. 1. The mean difference (bias) between PDFF-D and PDFF-H was 1.15%, and the limits of agreement were − 4.44 to 6.75%. Some points were outside the 95% limits of the agreement. ICC showed a good consistency (ICC 0.752, *P* < 0.01) between PDFF-D and PDFF-H.

**Figure 2:**
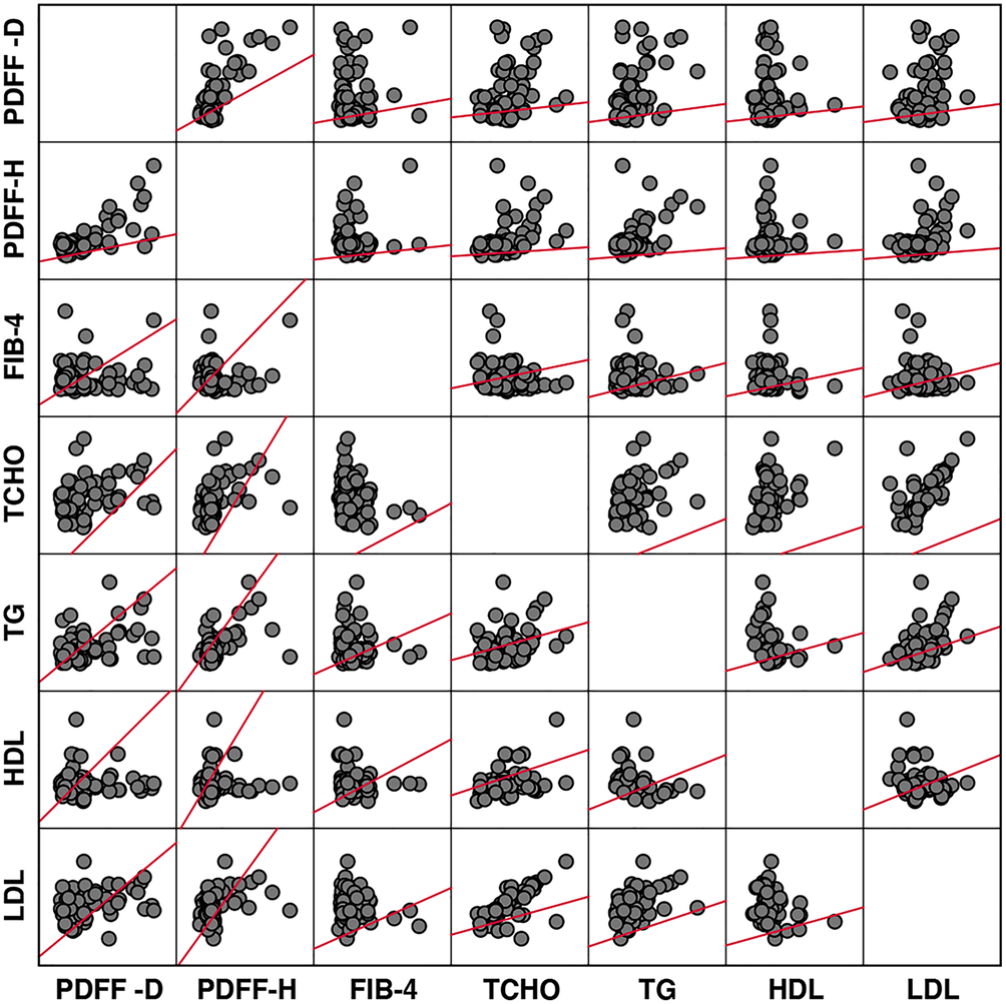
A scatterplot matrix with linear regression, associating the fat content measured by the breath-hold single-voxel high-speed T2-corrected multi-echo 1H MRS (HISTO) and multi-echo Dixon (ME-Dixon) sequences with the blood serum parameters. *PDFF-D* proton density fat fraction of ME-Dixon, *DPDFF-H* proton density fat fraction of HISTO, *FIB-4* Fibrosis-4 index, *TCHO* total cholesterol, *TG* triglyceride, *HDL* high-density lipoproteins, *LDL* low-density lipoproteins.

The relationships between PDFF-D and PDFF-H and the blood serum parameters are displayed as a scatter plot matrix in Fig. 2. The best indicators were those separating the clusters in an optimal way, with no overlapping in the scatter plot.

Table 1 shows mild-to-moderate correlations between PDFF and the blood serum parameters (total cholesterol, triglyceride, and high- and low-density lipoproteins), somewhat higher in PDFF-H than in PDFF-D.

**Table 1:** Spearman correlation coefficients of PDFF-D and PDFF-H with the blood serum parameters.

Protocol	PDFF-D	PDFF-H	FIB-4	TCHO	TG	HDL	LDL	
PDFF-D	–	0.551*	− 0.185	0.314*	0.367*	− 0.003	0.265*	r
		< 0.001	0.128	0.009	0.002	0.983	0.028	*P*
PDFF-H	0.551*	–	− 0.261	0.410*	0.389*	− 0.050	0.313*	r
	< 0.001		0.030	< 0.001	0.001	0.685	0.009	*P*

*PDFF-D* proton density fat fraction of multi-echo Dixon, *PDFF-H* proton density fat fraction of breath-hold single-voxel high-speed T2-corrected multi-echo ^1^H MRS, *FIB-4* Fibrosis-4 score, *TCHO* serum total cholesterol, *TG* serum triglyceride, *HDL* high density lipoprotein, *LDL* low density lipoprotein.

*P value < 0.05.

**Figure 3:**
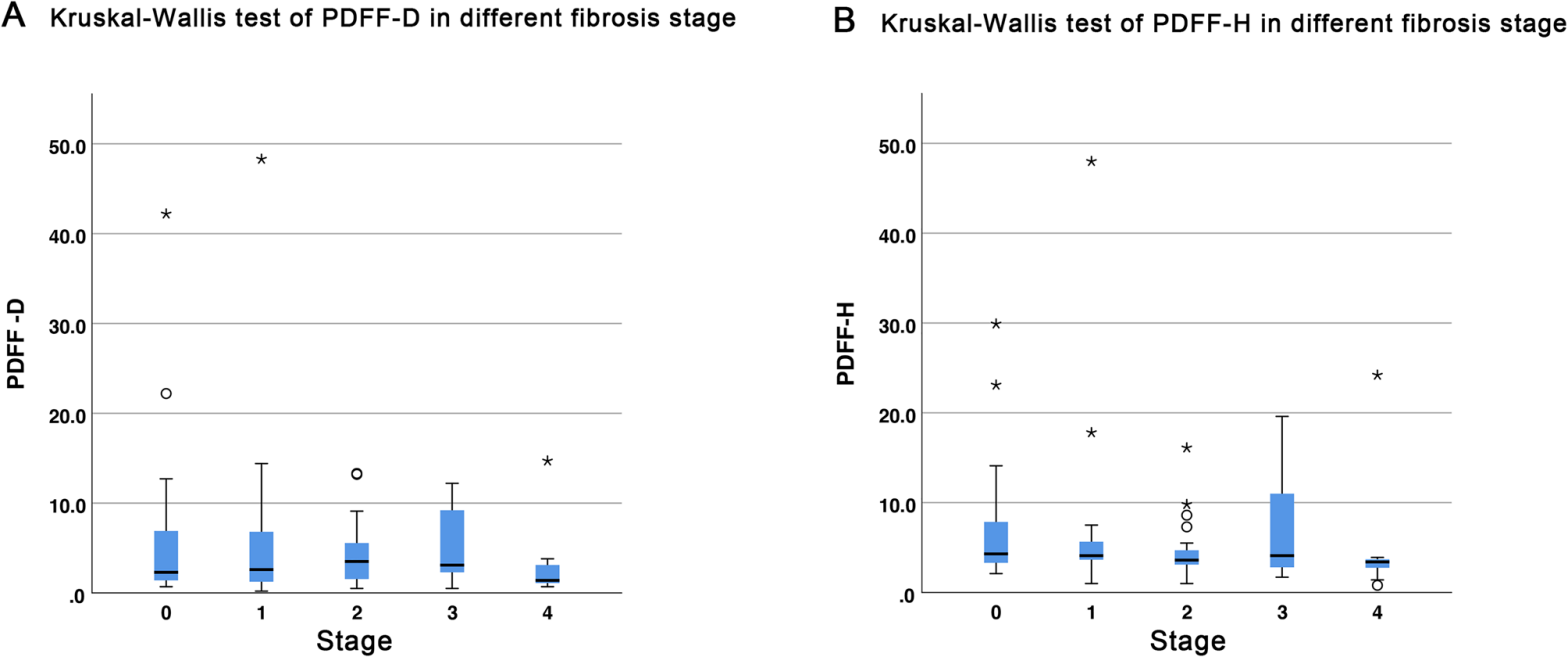
Kruskal–Wallis test to assess the difference between the fibrosis stage groups. Outlier data points marked by open circles were 1.5–3.0 interquartile ranges from the box ends. Outlier data points marked by asterisks were more than three interquartile ranges from the box ends. *PDFF* proton density fat fraction, *HISTO* breath-hold single-voxel high-speed T2-corrected multi-echo ^1^H MRS, *ME-Dixon* multi-echo Dixon, *PDFF-D* PDFF estimated by ME-Dixon, *PDFF-H* PDFF estimated by HISTO.

The PDFF and steatosis distributions for the healthy volunteers and patients in the various fibrosis stages are shown in Table 2. Both showed similar steatosis distribution among the various fibrosis stages. The Kruskal–Wallis test found no difference among the fibrosis stage groups (Fig. 3). The correlations between PDFF and the fibrosis stage (S) and inflammation activity (G) in the biopsies are shown in Table 3. A mild negative correlation was found between PDFF-H and the fibrosis stages (r = − 0.220, *P* = 0.04).

**Table 2:** PDFF-D and PDFF-H means and SDs and steatosis of healthy volunteers (S0) and patients with fibrosis staged 1–4 (S1–4).

	S0	S1	S2	S3	S4	*F*	*P*
*n*	24	15	27	10	11		
PDFF-D	5.81 ± 9.18	6.99 ± 12.19	4.14 ± 3.57	5.11 ± 4.11	2.98 ± 4.03	3.075	0.545
PDFF-H	6.98 ± 6.76	8.03 ± 11.69	4.56 ± 2.98	7.37 ± 5.96	4.84 ± 6.50	7.018	0.135
Steatosis(n)	7	4	3	4	1		0.211

*PDFF-D* proton density fat fraction of multi-echo Dixon, *PDFF-H* proton density fat fraction of breath-hold single-voxel high-speed T2-corrected multi-echo ^1^H MRS.

**Table 3:** PDFF-D and PDFF-H Spearman’s rank correlations with the fibrosis stages (S) and inflammation activity (G) in the biopsies.

Protocol	PDFF-D	PDFF-H	S	G	
PDFF-D	–	0.664*	− 0.035	0.035	r
	–	< 0.001	0.747	0.745	*P*
PDFF-H	0.664*	–	− 0.220*	–0.120	r
	< 0.001	–	0.040	0.266	*P*

*PDFF-D* proton density fat fraction of multi-echo Dixon, *PDFF-H* proton density fat fraction of breath-hold single-voxel high-speed T2-corrected multi-echo ^1^H MRS.

*P value < 0.05.

## Discussion

NAFLD, a common liver steatosis condition, is a complex disease spectrum, ranging from steatosis to non-alcoholic steatohepatitis and fibrosis^10^. Significant fibrosis is an important histological feature of non-alcoholic steatohepatitis, an aggressive form of this disease^11^. Therefore, liver fat content and fibrosis are closely associated and important for the disease prognosis.

Liver biopsy is currently the gold standard method to assess liver fibrosis and fat content in clinical practice. However, it is invasive and associated with sampling and interpretation errors, potential complications, and poor patient acceptance, restricting its wide clinical application^12, 13^. Noninvasive methods, especially quantitative imaging ones, receive increasing attention and use in clinical monitoring of liver fat content^14^ and liver fibrosis staging^15–17^.

This study investigated two MRI-based methods to quantify liver fat content and probe its impact on liver fibrosis staging.

### Quantification of liver fat with ME-Dixon and HISTO sequences

PDFF is a fundamental property of tissues, reflecting their fat concentration. PDFF is increasingly recognized as an accurate, noninvasive liver fat detection and quantification method^18^.

ME-Dixon and HISTO sequences are frequently used to quantify liver fat content in general practice. ME-Dixon sequence is a variant of the modified chemical shift-encoded MRI method, which provides accurate lipid fraction and, thus, fat content quantification in the entire liver. HISTO sequence is a fast breath-hold MRS method for T2-corrected liver lipid measurement. It overcomes the accuracy and speed limitations of the conventional ^1^H MRS. Both ME-Dixon and HISTO sequences allow for acquisition within a single breath-hold cycle.

Both PDFF-D and PDFF-H showed significant correlation with TCHO, TG and LDL. Which provide the potential value as biomarker of liver fat content in clinical medicine. Like some previous studies^19–23^, we observed a significant correlation between PDFF-D and PDFF-H (ICC 0.752, *P* < 0.01) even though the Bland–Altman plot showed a limit to the agreement between PDFF-D and PDFF-H, and ICC showed a good consistent between them. There could be several reasons for our correlation result being weaker than the strong correlation (r = 0.984 and 0.986) observed between chemical shift-encoded-PDFF and MRS-PDFF in previous studies^20, 22^. First, HISTO is a single-voxel spectroscopy acquisition mode, obtained in this study using a VOI placed in the right posterior liver segment. However, it is known that fat distribution in the liver is often heterogeneous. Consequently, sampling errors may have impacted our HISTO results. Second, 63 of the 75 patients in our study had a liver fat content lower than 5%. Pineda et al.^24^ reported that the signal-to-noise ratio decreases with the decrease in the liver fat content. This may have affected our ability to accurately quantify the liver fat content by the ME-Dixon and HISTO sequences.

The similar results were also seen in the research of Zhong et al.^25^ and Bashir et al.^26^. They pointed that MRI-based measure of liver PDFF are clinically feasible and accurate. The difference is that they both based on 3.0 T scan, while our study was based on 1.5 T scan. These maybe a testament of MRI-based measurements of PDFF across a variety of platforms.

### The impact of liver fat on liver fibrosis staging

Liver fibrosis is an early stage of liver damage. It is a common pathological process in many chronic liver diseases. The initial stage is chronic liver inflammation, followed by diffuse liver fibrosis, replacement of the normal liver architecture by regenerative hepatic nodules, and eventually liver failure^27^. Liver fibrosis is the leading long-term outcome predictor among all the histopathological changes identified in chronic liver diseases^28^. Therefore, noninvasive liver fibrosis assessment has always been the focus of clinical research. Liver fibrosis staging is influenced by complex histopathological changes such as steatosis and inflammation. The relationship between steatosis and fibrosis has attracted our attention. We defined steatosis as PDFF > 5%, and so also PDFF-D and PDFF-H > 5%. Nevertheless, we found no association between the various fibrosis stage groups and PDFF-D, PDFF-H, or steatosis. Spearman correlation showed that PDFF-H was weakly and negatively correlated with the fibrosis stages (r = − 0.220, *P* = 0.04). All these may suggest that the liver fat content has no direct effect on liver fibrosis stage similar with the previous study^29^. It was different from Ajmera et al.^30^, their research suggested a clear link between steatosis and fibrosis severity. The reasons for this may because: first, in their study, the number of patients with lower and higher liver fat content was similar. While, in our study most participants had low liver fat content. Second, the subject of their research was NAFLD, but our study patient population consist a large extent of HBV. Further studies will be needed to validate these findings.

We found through a literature review that a “two-hit” hypothesis was established as early as in 1998^31^. Steatosis is just the first “hit” on the liver. Whether patients with steatosis progress to necroinflammation or fibrosis depends on the presence of some other factors, known as the second “hit.” This could be a plausible endorsement for our findings. This ‘two-hit’ hypothesis has further evolved into the ‘‘multiple-hit” hypothesis based on novel findings from recent research^32^. In addition to steatosis, other factors, including insulin resistance, inflammatory mediators, lipopolysaccharides, and genetic factors, drive the liver parenchyma toward fibrosis.

### Limitations

This study had some limitations. First, we did not compare the MRI-based PDFF with semi-automatic histological fat quantification. Liver biopsies are usually done at our hospital to determine the etiology, so the pathologists ignore the fat content. Furthermore, liver biopsies have inherent sampling errors, especially when assessing liver fat content. Second, we did not divide the patients based on the etiology. There are five causes in our patients in this research (HBV, NAFLD, chronic liver disease without hepatitis virus infection, drug toxicities, and autoimmune). We found that in group of NAFLD, the PDFF was higher than other groups’ (*P* < 0.05), the difference was statistically significant, and the difference between other groups has no significant. This may have added to the confounding factors in this study and should be improved in the future. Third, as a single-voxel protocol, HISTO should be sampled in multiple sites, averaging the results. Such an approach could solve the sampling error issue and improve the accuracy; however, we sampled only one site in this study. One side, the single sampling method was used to increase the comparability of these two methods. And they were placed separately at the scanning by the same physician. On the other hand, the manual operation was really difficult to ensure the absolute consistency of the two mining positions. This may affect the consistency of the two methods. In future clinical application, multi-point sampling is still recommended.

Future work is needed to assess a larger number of patients in one etiology and to correlate PDFF with histological findings. For better comparison with ME-Dixon, we only chose one sample site of HISTO. Applied as a fat quantification method to the clinic, we recommend multiple sites of HISTO.

## Methods

### Study design and patients

This prospective study was conducted in accordance with the guidelines of the Declaration of Helsinki for Human Research and approved by the ethics committee of Lanzhou University Second Hospital (2020A-236), and all participants provided written informed consent.

**Figure 4:**
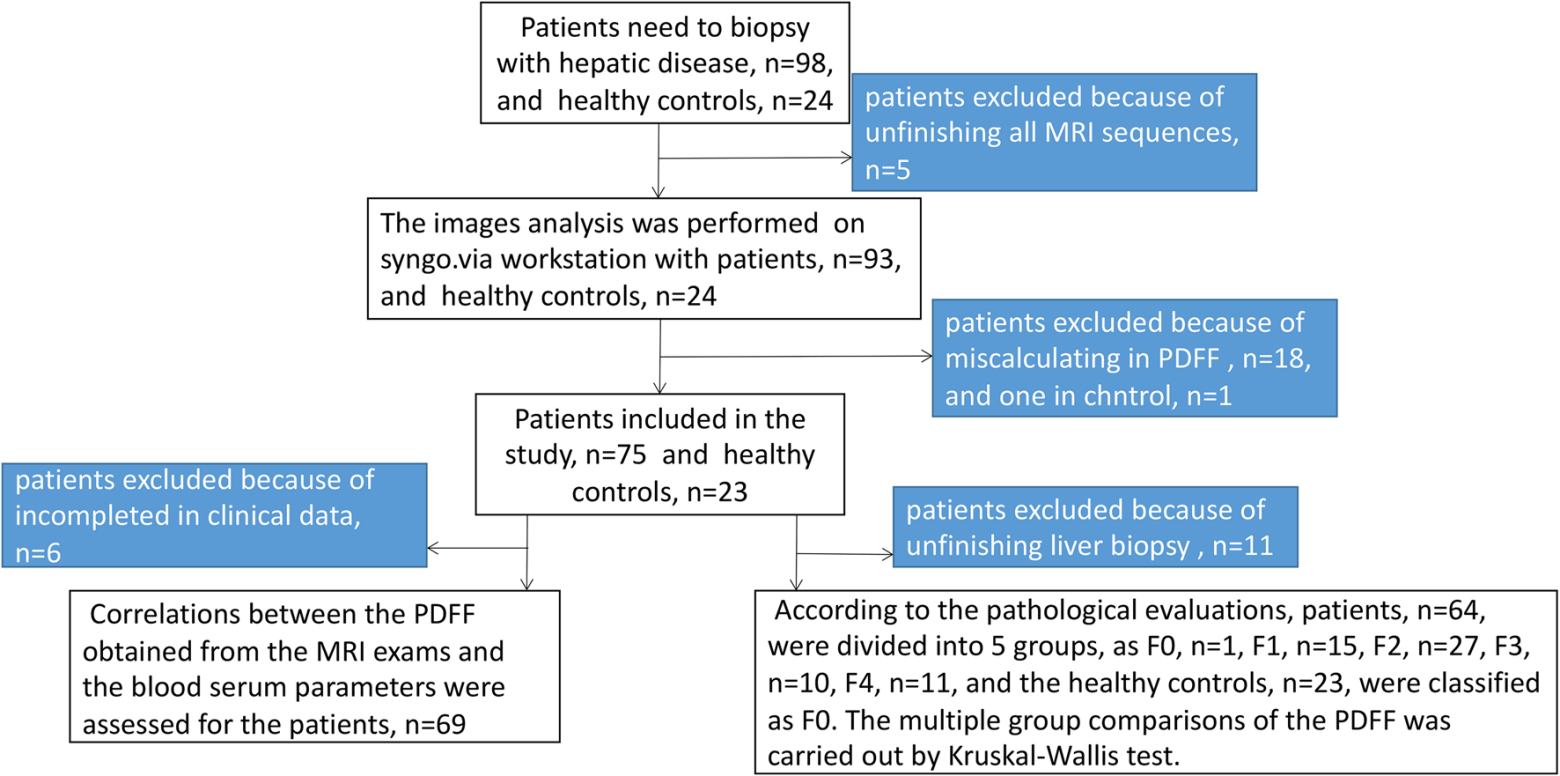
Study patient enrollment flow chart. *PDFF* proton density fat fraction.

This study enrolled 98 patients with chronic liver diseases and 24 healthy adult volunteers without serious health problems from Oct 2020 to June 2022. The inclusion criterion for the patients was adults with a chronic liver disease scheduled to undergo a liver biopsy. The inclusion criterion for the healthy volunteers was adults without serious health problems. The exclusion criteria included contraindications to MRI (claustrophobia, metal implants, or pacemakers). Figure 4 summarizes the participant recruitment process for the study. The underlying causes of the chronic liver disease in the 75 patients were hepatitis B (*n* = 56), nonalcoholic steatohepatitis (*n* = 8), a chronic liver disease without hepatitis virus infection (*n* = 6), drug toxicities (*n* = 4), and autoimmune (*n* = 1). Liver fibrosis was confirmed by biopsy in 64 patients. The liver biopsy and MRI were performed within a one-week interval.

### MRI examination

**Figure 5:**
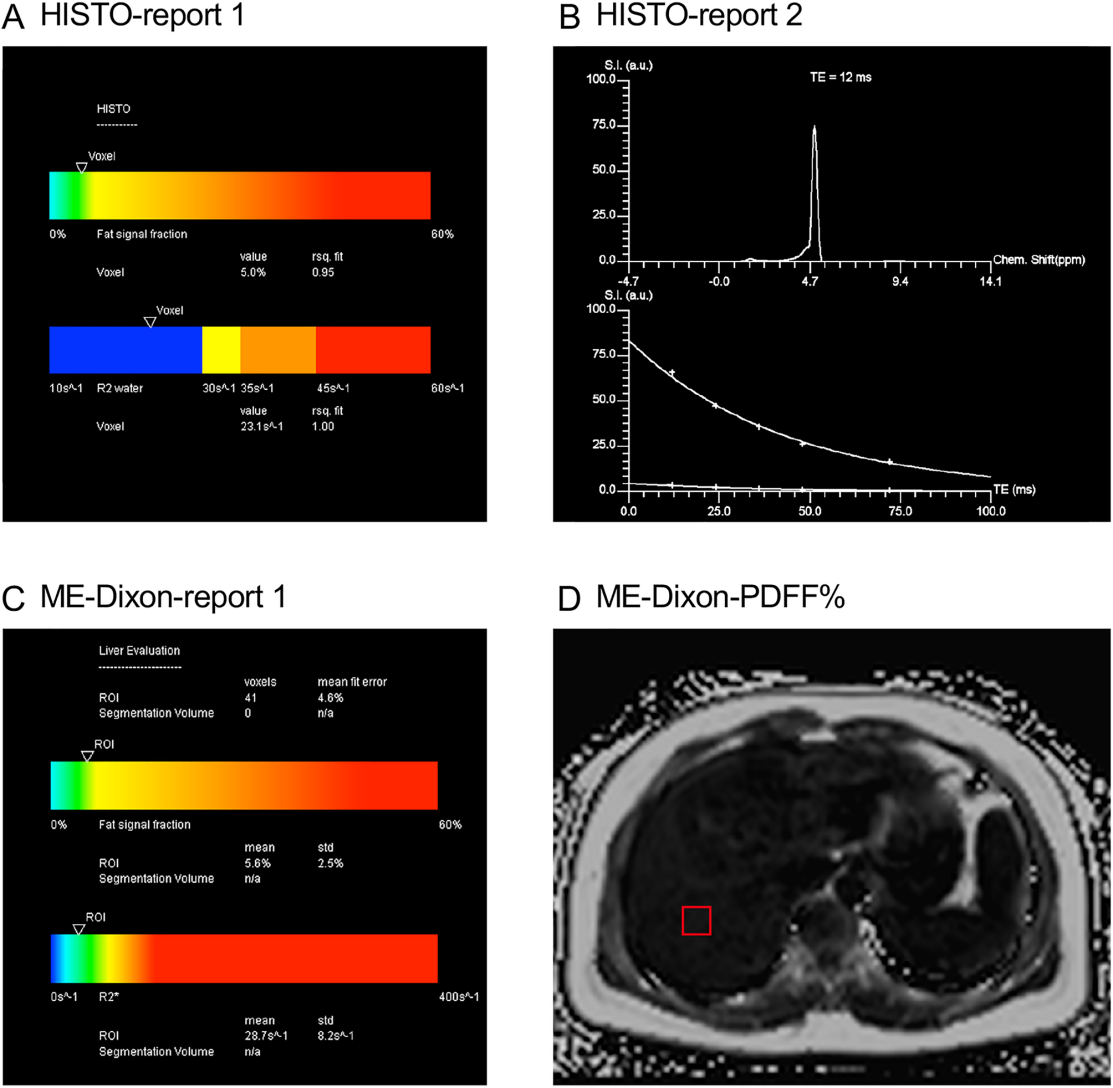
An example of the ME-Dixon and HISTO sequences. Multi-echo Dixon (ME-Dixon) and breath-hold single-voxel high-speed T2-corrected multi-echo 1H MRS (HISTO) sequence images of a 37-year-old female patient with chronic hepatitis B and G2S2 liver fibrosis in histology. The HISTO and ME-Dixon sequence reports show a proton density fat fraction (PDFF) of 5.0 and 5.6%, respectively. D is the slice corresponding to the report in C, and the red squares indicate the placement of the region of interest.

MRI examination was performed on a 1.5 T MR scanner (MAGNETOM Aera, Siemens Healthineers, Erlangen, Germany) using a combination of 18-channel body and 32-channel spine matrix coil elements. The participants underwent MR scanning using the ME-Dixon and HISTO protocols in the LiverLab package (Siemens Healthineers). An axial ME-Dixon sequence was performed with the following parameters: TR = 15.6 ms; TE = 2.38, 4.76, 7.14, 9.52, 11.9, and 14.28 ms, flip angle = 4°, FOV = 450 × 394 mm; slice thickness = 3.5 mm, voxel size = 1.4 × 1.4 × 3.5 mm, number of slices = 64. The HISTO sequence was performed with the following parameters: TR = 3,000 ms, TE = 12, 24, 36, 48, and 72 ms, flip angle = 90°, VOI = 30 × 30 × 30 mm, scan time is 15 s. We selected the right liver lobe (hilar level) near the ME-Dixon sequence ROI for the HISTO sequence, avoiding bile ducts and blood vessels. Figure 5 shows an example of the ME-Dixon and HISTO sequences. Subsequently, we calculated the proton density fat fraction (PDFF), defined as the fat-to-total (fat and water) signal strength ratio^33^, and named PDFF-D and PDFF-H, respectively.

### Biochemical tests and liver biopsies

One clinical medicine expert reviewed the patients’ medical records. Blood serum parameters were recorded within one week before or after the MRI examination. The participants’ demographic and clinical characteristics are presented in Table 4. The Fibrosis-4 index was calculated based on clinical and routine laboratory variables using previously-defined algorithms^34^. Histopathologic assessments were performed by an experienced pathologist, who graded liver fibrosis (F0–F4) based on the Scheuer semiquantitative scoring system^35^.

**Table 4:** Demographic and clinical data of the study population.

Parameter	Patients	Healthy control
No. of participants	75	23
Sex (male:female)	40:35	11:12
Mean age (years)	40 ± 11	39 ± 13
Body mass index, BMI (kg/m^2^)	23.81 ± 3.05	22.98 ± 2.95
AST level (U/L)	46.19 ± 63.97 (*n* = 74)	–
ALT level (U/L)	56.53 ± 72.22 (*n* = 74)	–
Serum albumin (g/L)	42.40 ± 4.93 (*n* = 74)	–
Serum total cholesterol, TCHO (mg/L)	38.31 ± 9.12 (*n* = 69)	–
Serum triglyceride, TG (mg/L)	15.19 ± 9.67 (*n* = 69)	–
High-density lipoprotein, HDL (mmol/L)	1.30 ± 0.63 (*n* = 69)	–
Low-density lipoprotein, LDL (mmol/L)	2.40 ± 0.86 (*n* = 69)	–

*AST* aspartate aminotransferase, *ALT* alanine aminotransferase.

### Statistical analysis

Agreement between fat content calculated from the ME-Dixon and HISTO sequences and blood serum parameters was assessed in three steps. First, we estimated the agreement using Bland–Altman plots, in which the difference between PDFF-D and PDFF-H was plotted against their means with their 95% limits of agreement. Subsequently, the intraclass correlation coefficient (ICC) assessed the degree of agreement between PDFF-D and PDFF-H. Second, we drew a scatterplot and performed linear regression to assess the association between the PDFF-D or PDFF-H and the blood serum parameters. Third, we used Spearman correlation coefficients to characterize the association of PDFF-D or PDFF-H measures and the blood serum parameters.

The Kruskal–Wallis test evaluated the distribution of PDFF-D and PDFF-H among the liver fibrosis stages. Fisher’s exact probabilities tested steatosis distribution among the liver fibrosis stages. PDFF-D and PDFF-H were defined as steatosis when > 5%. The Spearman’s rank correlation coefficients assessed the correlation between PDFF and the fibrosis stages.

Statistical analysis was performed using IBM SPSS Statistics for Windows, Version 26.0 (IBM Corp., Armonk, NY, USA), and R statistical software version 4.0.3 (R Foundation for Statistical Computing, Vienna, Austria). Two-sided *P* values < 0.05 were considered statistically significant.

## Conclusions

ME-Dixon and HISTO sequences are noninvasive MR imaging methods for measuring liver fat content. The liver fat content showed no direct impact on the liver fibrosis stages.

## Data Availability

Availability of data and materials. All data generated and analyzed during the current study will be available from the corresponding author on reasonable request.
